# Relation of resting brain signal variability to cognitive and socioemotional measures in an adult lifespan sample

**DOI:** 10.1093/scan/nsad044

**Published:** 2023-09-12

**Authors:** Cheryl L Grady, Jenny R Rieck, Giulia Baracchini, Brennan DeSouza

**Affiliations:** Rotman Research Institute at Baycrest, Toronto, Ontario M6A 2E1, Canada; Departments of Psychiatry and Psychology, University of Toronto, Toronto, Ontario M5S 3G3, Canada; Rotman Research Institute at Baycrest, Toronto, Ontario M6A 2E1, Canada; Department of Neurology and Neurosurgery, Montréal Neurological Institute, McGill University, Montréal, Quebec H3A 0G4, Canada; Rotman Research Institute at Baycrest, Toronto, Ontario M6A 2E1, Canada

**Keywords:** social, emotion, cognition, variability, fMRI

## Abstract

Temporal variability of the fMRI-derived blood-oxygen-level-dependent (BOLD) signal during cognitive tasks shows important associations with individual differences in age and performance. Less is known about relations between spontaneous BOLD variability measured at rest and relatively stable cognitive measures, such as IQ or socioemotional function. Here, we examined associations among resting BOLD variability, cognitive/socioemotional scores from the NIH Toolbox and optimal time of day for alertness (chronotype) in a sample of 157 adults from 20 to 86 years of age. To investigate individual differences in these associations independently of age, we regressed age out from both behavioral and BOLD variability scores. We hypothesized that greater BOLD variability would be related to higher fluid cognition scores, more positive scores on socioemotional scales and a morningness chronotype. Consistent with this idea, we found positive correlations between resting BOLD variability, positive socioemotional scores (e.g. self-efficacy) and morning chronotype, as well as negative correlations between variability and negative emotional scores (e.g. loneliness). Unexpectedly, we found negative correlations between BOLD variability and fluid cognition. These results suggest that greater resting brain signal variability facilitates optimal socioemotional function and characterizes those with morning-type circadian rhythms, but individuals with greater fluid cognition may be more likely to show less temporal variability in spontaneous measures of BOLD activity.

## Introduction

There has been increasing interest in the past decade on examining temporal brain signal variability, defined as the deviation in the functional magnetic resonance (fMRI), electroencephalography and magnetoencephalography brain signals over time, and how it relates to cognition. Much of this work has assessed the association between fMRI-derived blood-oxygen-level-dependent (BOLD) signal variability measured during a cognitive task and performance on that task. Such work has generally found that increasing brain signal variability in a wide set of regions is associated with more accurate and/or more consistent performance (for a review, see Waschke *et al.*, 2021). These studies have provided important insights into how different types of cognitive processing are supported by brain signal variability. Evidence to date suggests that temporal brain signal variability is an intrinsic characteristic of optimal neural functioning, which may allow for the efficient processing of incoming information (Faisal *et al.*, 2008; McIntosh *et al.*, 2010; Padmanabhan and Urban, 2010).

Measures of brain signal variability obtained from resting scans also have been examined in healthy participants (Nomi *et al.*, 2017; Grady and Garrett, 2018; Baracchini *et al.*, 2021) and those with various disorders (Nomi *et al.*, 2018; Kebets *et al.*, 2021). There is evidence that resting fMRI measures are stable over days or months (Poldrack *et al.*, 2015; Ge *et al.*, 2017) and that functional brain networks are dominated by stable individual factors rather than cognitive variation (Gratton *et al.*, 2018), suggesting important individual differences in spontaneous brain activity measured at rest that might reflect differences in cognition (Geerligs *et al.*, 2015). Taken together with the work on task-related fMRI, this evidence would suggest that brain signal variability during a particular cognitive task would be tightly linked to performance on that task, i.e. that it would reflect the specific cognitive demands at that point in time. On the other hand, resting BOLD-SD might be more strongly related to behavioral characteristics that are relatively stable over time, such as fluid or crystallized cognition, or socioemotional self-reports. If brain signal variability is indeed an intrinsic characteristic of optimal neural functioning and facilitates neural information transfer between regions (Baracchini *et al.*, 2021), then one might expect its behavioral relevance to extend beyond externally driven task performance to more general behavior as well. In this study, we tested this hypothesis by assessing the relation between the standard deviation (s.d.) of the resting BOLD signal (i.e. BOLD variability via BOLD-SD) and cognitive and socioemotional measures from the NIH Toolbox. We also investigated the association between BOLD variability and optimal time of day for alertness (chronotype) because this characteristic is known to influence both cognition and emotion.

### Cognitive measures

In this study, we examined two composite measures of cognitive function from the NIH Toolbox, i.e. fluid intelligence (gF) that combines scores on tests that engage flexible thinking, such as the flanker task and list sorting, and crystallized intelligence (gC), which characterizes tasks relying on acquired knowledge, such as vocabulary and reading comprehension (Cattell and Kuhlen, 1963). Decreased reaction time, increased stability of responding and better accuracy on a variety of tasks requiring flexible cognition are associated with greater task-related BOLD-SD in a number of studies (Garrett *et al.*, 2011, 2021; Guitart-Masip *et al.*, 2016; Grady and Garrett, 2018; Malins *et al.*, 2018; Good *et al.*, 2020), although not all (Boylan *et al.*, 2021). Consistent with the bulk of this work, we recently reported that faster and more accurate working memory performance was associated with increased task-related BOLD-SD in attention and cognitive control brain networks, as well as reduced BOLD-SD in sensorimotor networks (Rieck *et al.*, 2022).

In terms of resting BOLD, some studies have found that characteristics of resting activity are related to cognitive performance. Finn *et al.* (2015) showed that individually derived profiles of resting functional connectivity in the frontoparietal cortex are correlated with gF as measured with Raven’s Progressive Matrices (RPM; Raven *et al.*, 1976) in the Human Connectome (HCP) dataset of young adults, suggesting that interindividual variability across adults carries information about cognitive characteristics that are relatively stable over time. Using resting multiscale entropy (MSE), a measure of within-subject temporal complexity that is similar to BOLD variability, another study (Omidvarnia *et al.*, 2021) found that higher MSE across eight brain networks, including the default mode network (DMN), frontoparietal control network and the two attention networks (dorsal and ventral), was associated with higher scores on the RPM measure of gF in the HCP dataset. Using BOLD-SD, a recent study (Garrett *et al.*, 2021) examined the associations among gF as assessed by the Cattell Culture Fair Intelligence Test (Cattell and Cattell, 1973), gC (vocabulary) and intra-person BOLD variability during rest in a sample of adults from 19 to 82 years of age. Both gF and working memory were positively correlated with BOLD variability in widespread cortical areas, age was negatively correlated with variability in these regions and neither gC nor speed measures showed a robust correlation. In contrast, Millar *et al.* (2020) found that resting BOLD variability in middle-aged and older adults was not related to a composite measure of global cognition derived from scores across multiple cognitive domains (i.e. semantic fluency, processing speed, executive function and free recall) after adjusting for age. These studies suggest that resting measures of spontaneous brain activity, including BOLD-SD, are more likely to be related to performance on cognitive tests that assess gF than those tapping into gC, but also that the specific cognitive processes of interest and the age of the participants may be important factors in the association of resting BOLD with composite measures of cognition.

### Socioemotional measures

The processing of socioemotional stimuli involves many brain regions, including the amygdala, ventral striatum and ventral frontal cortex (Gobbini *et al.*, 2004; Bickart *et al.*, 2012; Farkas *et al.*, 2021; Merritt *et al.*, 2021; Kim *et al.*, 2022). Recent work has shown that resting DMN functional connectivity is related to greater life satisfaction (Smith *et al.*, 2015) and socioemotional adaptation (Petrican *et al.*, 2021) and that reduced activity of the retrosplenial cortex (Li *et al.*, 2020), a node of the DMN, is related to higher fear-affect scores on the NIH Toolbox scale. Other studies have shown that within-subject measures of the amplitude of resting activity [the so-called amplitude of low-frequency fluctuations (ALFF)] are related to socioemotional measures such as sensitivity to rejection (Sun *et al.*, 2018), self-esteem (Pan *et al.*, 2016) and self-control (Li *et al.*, 2021). In terms of variability measures, both major depressive disorder and generalized anxiety disorder are accompanied by greater variability of dynamic ALFF in both cortical and subcortical regions, compared to healthy controls (Cui *et al.*, 2020; Zheng *et al.*, 2021). Similarly, individuals with depression or borderline personality disorder who show emotional lability also have greater resting BOLD-SD in the amygdala and ventromedial prefrontal cortex (PFC) than controls (Kebets *et al.*, 2021), and resting BOLD-SD obtained prior to treatment (Mansson *et al.*, 2022) can predict good treatment outcome in patients with social anxiety disorder. Although these data from patient groups would suggest that greater BOLD variability is associated with poorer socioemotional function, it remains unclear how BOLD-SD might relate to measures of socioemotional function in healthy adults. It is possible that the relation between BOLD variability and socioemotional function follows an inverted U-shaped function, such that an optimal level of variability might facilitate socioemotional adaptation, with both lower and higher levels accompanying poorer function. This could account for the differences between patient groups and controls. This scenario, along with the evidence that brain signal variability is beneficial for cognitive performance, would suggest that better socioemotional function in the ‘normal range’ would be associated with increased resting BOLD variability. In addition, the positive relation reported in the literature between gF and successful socioemotional function (Huepe *et al.*, 2011; Schlegel *et al.*, 2017) suggests that increased BOLD variability might facilitate performance on both cognitive and socioemotional measures.

### Chronotype

Chronotype refers to the preferred time of day when people feel most alert, which can be the morning, middle of the day or evening (Horne and Ostberg, 1976). Much of the literature on cognition and chronotype deals with the synchrony effect, which refers to the finding that morning people perform better on cognitive tasks in the morning, whereas evening people perform better in the evening, particularly on tasks requiring cognitive control (Hahn *et al.*, 2012; Lara *et al.*, 2014). In contrast, more automatic processes, such as encoding unattended material (Rothen and Meier, 2016) and implicit learning (Delpouve *et al.*, 2014), are enhanced at off-peak times. Some overall differences between chronotypes also have been reported. For example, evening types have higher narcissism (Zajenkowski *et al.*, 2019), are at higher risk for depression or report more symptoms of depression (Hidalgo *et al.*, 2009; Au and Reece, 2017; Kivela *et al.*, 2018; Bauducco *et al.*, 2020; Kim *et al.*, 2020; Lunsford-Avery *et al.*, 2021; Wills *et al.*, 2021; Gorgol *et al.*, 2022) and report more symptoms of ADHD (Korman *et al.*, 2019), alcohol use disorder (Taylor *et al.*, 2020), perceived stress (You *et al.*, 2020) and anxiety (Cox and Olatunji, 2019). Evening types also have poorer socioemotional function (Lunsford-Avery *et al.*, 2019), poorer emotional regulation (Ottoni *et al.*, 2012; Watts and Norbury, 2017) and lower happiness (Tan *et al.*, 2020). In contrast, morningness is associated with greater life satisfaction and conscientiousness (Drezno *et al.*, 2019) and more positive mood (Biss and Hasher, 2012).

Most of the MRI studies of chronotype have focused on synchrony and find more activation in task-related areas when individuals are scanned at their optimal time of day for tasks such as inhibition (Schmidt *et al.*, 2012; Anderson *et al.*, 2014; Song *et al.*, 2018) and working memory (Schmidt *et al.*, 2015). In terms of group differences, results are mixed. Resting functional connectivity in the DMN was found to be greater in morning *vs* evening chronotypes in some studies (Horne and Norbury, 2018a; Facer-Childs *et al.*, 2019), but lower in morning types in another study (Tian *et al.*, 2020). Evening chronotype has been associated with enhanced amygdala response to fearful faces and reduced functional connectivity between the amygdala and the anterior cingulate cortex (Horne and Norbury, 2018b), as well as more activity in sensorimotor areas during a semantic priming task (Rosenberg *et al.*, 2015). No studies to date have examined chronotype and BOLD-SD.

### Current study

In this study, we adopted a multivariate approach to assess the relations among resting BOLD-SD, cognitive composites and emotional measures from the NIH Toolbox, and chronotype in a sample of healthy adults. We expected to find brain regions where greater BOLD variability is associated with better cognition, better socioemotional function and morning chronotype, based on prior work suggesting that greater BOLD variability would be beneficial to cognitive and socioemotional function. Specifically, we expected that greater BOLD variability would be associated with higher gF, but not necessarily higher gC scores, because gF reflects flexible and effortful cognition. We also hypothesized that greater BOLD variability would be associated with higher scores on indices of successful socioemotional function, and lower scores on negative mood/function measures, consistent with the idea that more neural variability is beneficial for optimal function within the normal range of socioemotional function. Finally, we expected that greater BOLD variability would be associated with higher morningness scores and greater synchrony between chronotype and time of testing, due to the association between morningness and better socioemotional function, and the typical finding that greater synchrony is accompanied by better cognitive control. In terms of brain regions, we expected that those regions involved in cognitive control and/or attention (e.g. nodes of the frontoparietal control and attention networks) as well as those underlying emotional functions (e.g. the amygdala) would show a positive association between greater variability and better cognitive/socioemotional function.

## Methods

### Participants

This study included 158 adults from the greater Toronto area (aged 20–86 years) and involved two testing sessions (cognition/emotion testing on the NIH Toolbox and MRI scanning), scheduled about 1 week apart. The sample included healthy (i.e. free from any major psychiatric or neurological conditions; no history of head trauma), cognitively normal (Mini Mental State Exam scores > 26; Folstein *et al.*, 1975), right-handed and fluent English speakers, with normal or corrected-to-normal vision (at least 20/30). If necessary, vision was corrected using MRI-compatible lenses during scanning. One subject (a 21-year-old female) fell asleep during the resting scan and was therefore excluded from the analyses. As such, the present study included a sample of 157 participants [mean age = 49.46, s.d. = 18.88] that was 62.42% female and had 16.95 years of education on average (s.d. = 2.87, Table 1).

**Table 1.** Sample demographics and task scores

Age group	20–31	32–48	49–65	66–86	Total sample	Correlation with age
*N*	41	36	42	38	157	
Females	23	22	29	24	98	
Education	16.95 (2.64)	17.31 (2.49)	17.31 (3.63)	16.22 (2.50)	16.95 (2.88)	
MMSE	29.07 (1.17)	29.36 (0.83)	28.81 (0.92)	28.53 (1.08)	28.94 (1.05)	−0.27**
Crystallized	122.70 (9.98)	127.17 (13.03)	128.70 (13.96)	131.38 (12.50)	127.43 (12.73)	0.25**
Fluid	121.71 (14.56)	113.39 (10.85)	102.67 (9.49)	93.60 (5.97)	107.90 (15.07)	−0.72 **
Instrumental support	46.62 (8.87)	41.58 (8.80)	46.05 (11.26)	47.41 (11.06)	45.51 (10.23)	0.07
Emotional support	47.53 (11.63)	43.10 (7.66)	43.53 (11.57)	44.86 (8.36)	44.80 (10.13)	−0.11
Friendship	48.53 (8.48)	45.59 (8.69)	45.06 (9.58)	49.09 (8.72)	47.06 (8.98)	−0.01
Self-efficacy	46.41 (8.22)	46.69 (7.68)	49.97 (8.68)	49.79 (7.87)	48.24 (8.24)	0.21**
Positive affect	46.65 (9.36)	44.51 (8.82)	44.97 (6.83)	47.80 (6.59)	45.99 (8.01)	0.03
Meaning & purpose	46.55 (11.82)	46.34 (8.35)	48.81 (11.48)	47.28 (7.88)	47.28 (10.09)	0.05
General life satisfaction	49.57 (9.55)	49.12 (9.38)	50.66 (9.47)	53.63 (8.36)	50.74 (9.29)	0.17*
Perceived rejection	51.88 (10.97)	52.86 (9.69)	52.56 (10.71)	49.82 (9.16)	51.79 (10.17)	−0.04
Perceived hostility	51.34 (10.71)	51.54 (8.88)	51.46 (9.02)	49.31 (6.56)	50.93 (8.92)	−0.08
Loneliness	55.73 (9.62)	56.09 (9.63)	54.53 (8.84)	51.14 (8.31)	54.38 (9.23)	−0.16*
Fear-somatic arousal	49.27 (9.02)	50.81 (8.19)	48.00 (7.35)	47.34 (6.31)	48.81 (7.83)	−0.15
Fear-affect	55.29 (9.61)	55.29 (6.37)	54.92 (7.39)	54.09 (6.52)	54.90 (7.58)	−0.06
Anger-physical aggression	52.03 (9.60)	52.61 (8.35)	49.73 (7.13)	46.65 (6.04)	50.25 (8.16)	−0.29**
Anger-hostility	54.00 (8.86)	52.55 (9.53)	49.21 (10.11)	46.11 (7.16)	50.47 (9.43)	−0.34**
Anger-affect	49.17 (11.25)	49.66 (9.05)	49.25 (7.48)	47.51 (6.60)	48.90 (8.75)	−0.06
Sadness	49.24 (9.64)	50.33 (8.11)	49.00 (7.69)	48.17 (7.16)	49.17 (8.18)	−0.06
Stress	54.14 (11.31)	52.11 (8.49)	51.68 (8.77)	46.50 (9.41)	51.17 (9.90)	−0.25**
MEQ	47.71 (11.92)	49.42 (10.70)	55.19 (11.16)	59.76 (9.17)	53.02 (11.73)	0.45**
Synchrony	−0.256 (0.961)	−0.012 (0.743)	0.170 (0.734)	0.107 (0.685)	0.001 (0.801)	0.17*

Values in parentheses are s.d.

* *P* < 0.05;

** *P* < 0.01.

### MRI session and acquisition

A Siemens Trio 3T magnet at Baycrest Health Sciences was used to scan all participants. Following a 30 min practice session inside an MRI simulator, participants underwent a 1.5 hour MRI scan with the following sequences: T2-weighted FLAIR (TR = 4000 ms, TE = 466 ms, FOV = 258 mm, 176 slices, 256 × 256 acquisition matrix), 10 min BOLD resting scan, T1-weighted anatomical imaging, three BOLD fMRI tasks (go/no-go, *n*-back and task switching), diffusion weighted imaging and, if time permitted, arterial spin labeling. The present study examined brain variability metrics for the resting scan, during which participants kept their eyes open for 10 min, focusing on a white fixation cross in the center of a black screen. A camera within the scanner allowed the experimenter to monitor the participant’s eyes to ensure they remained awake and focused on the fixation cross.

High-resolution anatomical scans were produced using a T1-weighted MP-RAGE sequence in which 160 axial slices were collected with the following parameters: TR = 2000 ms, TE = 2.63 ms, FOV = 256 mm, 192 × 256 × 160 acquisition matrix and 1 mm^3^ isotropic voxel. BOLD fMRI data were collected using an echo-planar imaging sequence with 40 axial slices acquired parallel to the anterior–posterior commissure using the following parameters: TR = 2000 ms, TE = 27 ms, flip angle = 70^°^, FOV = 192 mm, 64 × 64 × 40 acquisition matrix and 3 mm^3^ isotropic voxels (with 0.5 mm gap). In total, 297 volumes were collected during the resting scan.

### Behavioral measures

#### Cognitive

Participants completed a series of cognitive tasks as part of the NIH Toolbox. Scores on five of these tests (Flanker, Dimensional Change Card Sort, Picture Sequence Memory, List Sorting and Pattern Comparison) were averaged to create a Fluid Cognition Composite score (gF), and then scaled scores were derived based on this new distribution (devised such that the mean was 100 and the s.d. was 15). A separate composite scaled score for Crystallized Cognition captured performance in Picture Vocabulary and Oral Reading Recognition tests (gC). For the analysis, we used age-unadjusted scaled scores for these composite measures and then regressed age out of all scores, so we could estimate the relation between cognitive scores and BOLD-SD across our lifespan sample without the confounding effects of age. We opted to use the age residuals from the unadjusted cognitive scores, rather than using the ‘fully adjusted’ scores automatically provided by the NIH Toolbox, because the latter scores also corrected for race/ethnicity and this information was not available for every participant (i.e. not every participant had NIH-adjusted scores available). Detailed descriptions of the tasks used to create the composite scores are available at https://www.healthmeasures.net/explore-measurement-systems/nih-toolbox/intro-to-nih-toolbox/cognition. Information on the validity and reliability of these cognitive measures can be found in Heaton *et al.* (2014).

#### Socioemotional

Participants also completed a series of emotional questionnaires from the NIH Toolbox using either a 5- or 7-point Likert scale. These surveys assessed seven positive aspects of socioemotional function (instrumental support, emotional support, friendship, self-efficacy, positive affect, meaning & purpose and general life satisfaction) and 10 negative aspects (rejection, hostility, loneliness, fear-somatic arousal, fear-affect, anger-physical aggression, anger-hostility, anger-affect, sadness and stress). The Toolbox-generated T-Score for each scale was used in the analysis, after regressing age out of each, as was done with the cognitive scores. The T-Scores were created in the same way as the cognitive scaled scores; however, the T-Scores from the emotional questionnaire had a mean of 50 and an s.d. of 10. For more information on the emotion measures from the Toolbox, see Salsman *et al.* (2013).

#### Morningness-Eveningness Questionnaire

Morningness-Eveningness Questionnaire (MEQ) is a 19-item self-assessment questionnaire that evaluates circadian rhythms and sleep patterns, assessing activity and alertness at certain times of the day (Horne and Ostberg, 1976). Item scores are added together, creating a global sum that represents the participant’s optimal time of day. Global scores are typically converted into a five-point scale of ‘definitely evening type’ (16–30), ‘moderately evening type’ (31–41), ‘neutral’ (42–58), ‘moderately morning type’ (59–69) and ‘definitely morning type’ (70–86). For the purposes of this study, global scores were analyzed rather than categorical classifications, and age was regressed out of MEQ scores prior to analysis.

A score reflecting the synchrony between chronotype and time of testing was computed by first determining the midpoint of the MRI resting scan session to represent ‘time of testing’, with AM/PM values converted into a 24-h scale. Resulting times were subtracted from 24 so that higher time of testing values reflected earlier (morning) test sessions, similar to high MEQ scores reflecting a morning chronotype. Both MEQ scores and time of testing values were then standardized using *Z*-scores, and the absolute difference between a participant’s MEQ score and time of testing was calculated. This absolute difference was subtracted from 1, yielding a final synchrony score, with higher scores indicating a better match between a participant’s test time and their optimal time of day. Age was regressed out of the synchrony scores prior to analysis.

### fMRI preprocessing

Operationally, the measure of BOLD variability that we and others have used is the ‘moment-to-moment’ variability of the BOLD signal after all ‘nuisance’ sources of noise, such as head motion, have been removed, to the extent that we are able to do so. Therefore, we used our previous work (Garrett *et al.*, 2011; Rieck *et al.*, 2022) to guide the preprocessing of functional data in the current study, which featured the following steps. First, the FMRIB Software Library (FSL) Brain Extract Tool was used to create participant-specific brain masks. To correct for motion, functional volumes across the time series were co-registered using FEAT in FSL. Volumes then underwent temporal detrending and bandpass filtering (0.01–0.1 Hz). Next, FSL’s melodic tool was used to compute independent components (ICs) and dimensionality was estimated by the Laplace method, allowing for denoising with FIX. In a ‘training set’ of 40 participants (age range 20–86; mean age = 51.2, s.d. = 19.5), the spatial maps and time series for each IC were visually inspected and hand-labeled as ‘noise’ if any of the following were apparent: time-series spiking, spatial ringing, spotty or sparse spatial distribution of signal, low-frequency signal drift (<0.009 Hz), high power in high frequencies (>0.13 Hz), susceptibility and flow artifacts or white matter/ventricle activation (for more detailed guidelines on the classification of ‘noise’ ICs, see Kelly *et al.*, 2010; Garrett *et al.*, 2014). The classification of noise ICs for the remaining participants (i.e. the ‘test set’) was conducted based on the 40-participant training set. Noise ICs were then regressed out of the time series for each individual. Ultimately, an average of 52% (range 20–78%) of ICs were classified as ‘noise’. Motion parameters and signal in tissue of no interest (white matter, vessels and cerebrospinal fluid) were also regressed out of the data, followed by smoothing with a 7 mm^3^ kernel, warping to Montreal Neurological Institute space and resampling to 4 mm^3^ isotropic voxels.

### Brain variability measures

After preprocessing, SPM’s Variability Toolbox (https://github.com/LNDG/vartbx) was used to compute the s.d. of the BOLD time series (BOLD-SD) across the 10 min resting scan. First, the rest scan was normalized so that the overall mean across the brain and the entire scan was 100. Then, for each voxel, the mean was subtracted and s.d. was calculated across the time series (Garrett *et al.*, 2010). We used the s.d. metric here, instead of others that have been used in fMRI research, such as the mean square successive difference (MSSD), to be consistent with our prior work (Garrett *et al.*, 2010, 2014; Rieck *et al.*, 2022), although s.d. and MSSD are highly correlated with one another (*r* > 0.90, see Garrett *et al.*, 2011; Baracchini *et al.*, 2021; Rieck *et al.*, 2022). Preprocessed BOLD-SD brain maps for each participant, along with corresponding behavioral scores, are available at https://osf.io/wgs7k/.

### Primary statistical analyses

In a single analysis, behavioral partial least squares (PLS; www.rotman-baycrest.on.ca/pls/) was used to examine associations between BOLD-SD at rest and scores from cognitive tasks, emotional questionnaires and the MEQ (McIntosh and Lobaugh, 2004; Krishnan *et al.*, 2011). Because of its strong associations with BOLD-SD, age was regressed out of BOLD-SD brain maps using SPM 12 (Rieck *et al.*, 2022). To account for residual motion effects, the mean frame displacement (FD) was also regressed out of the BOLD-SD brain maps (using SPM 12). Behavioral PLS was used to calculate the covariance between BOLD-SD brain maps and all behavioral metrics of interest. This brain–behavior covariance matrix underwent singular value decomposition, yielding a set of orthogonal latent variables (LVs) that maximally explained the relation between brain and behavior.

Significant LVs were identified through permutation (1000 resamples) of the singular values. PLS also produced a loading (or salience) per voxel, which represented the contribution of BOLD-SD in that voxel to each LV. These voxel saliences were used to compute ‘brain scores’ for each participant, indicating the degree to which a participant expressed the BOLD-SD pattern for a particular LV. To determine how performance contributed to a pattern of brain activity, correlations between participant brain scores and behavioral measures were calculated. Robust correlations were identified through 1000 bootstrap resamples (with replacement) of the correlation values to compute 95% confidence intervals (CIs) around the original correlation values. Correlation CIs that did not cross zero were considered to indicate significant correlations between the brain pattern and the behavioral measures. Significant voxels contributing to each LV were identified by a *t-*like statistic called the bootstrap ratio (BSR; McIntosh and Lobaugh, 2004; Krishnan *et al.*, 2011), which was computed through bootstrap resampling of voxel saliences. BSRs represent the ratio of a voxel’s salience to an estimate of its standard error generated through the resampling procedure (1000 resamples). A BSR cut-off of 2.5 and a cluster minimum of 15 voxels were used to determine which regions robustly contributed to each LV. All BSR maps reported here were created using MANGO (https://ric.uthscsa.edu/mango/).

To determine the contribution of a set of brain networks to the whole-brain maps of BOLD-SD, we identified functional networks using a 200-parcel atlas of seven brain networks (Yeo *et al.*, 2011; Schaefer *et al.*, 2018), including the visual (VIS), somato-motor (SomMot), dorsal attention (DAN), salience/ventral attention (SalVAN), limbic (LIM), fronto-parietal control (CON) and DMN. To provide a metric for each network’s contribution to the whole-brain results, the network atlas was overlaid with significant voxels from the PLS analysis, and the proportion of significant voxels to total voxels in each network was calculated. Because the cerebellum, amygdala, hippocampus and subcortical regions are not included in the seven networks of the Yeo/Schaefer atlas, we used the AAL atlas (Tzourio-Mazoyer *et al.*, 2002) to define three additional anatomical areas that would encompass these regions: bilateral cerebellum, bilateral medial temporal regions (MTL, including amygdala, hippocampus and parahippocampal gyrus) and bilateral subcortical structures (caudate, putamen, globus pallidus and thalamus).

## Results

### Behavioral measures

The mean scores on the NIH Toolbox tests and the MEQ are shown in Table 1, broken down into four age groups to illustrate the typical age effects in our sample (age was kept continuous in all analyses). As expected, gF was negatively correlated with age, whereas gC and MEQ were positively correlated with age (May and Hasher, 1998; Park and Reuter-Lorenz, 2009). In addition, some of the negative emotion measures were negatively correlated with age, and self-efficacy and life satisfaction were positively related to age, consistent with the ‘positivity bias’ that is often reported in older adults (Carstensen *et al.*, 2003; Mather and Carstensen, 2003). These correlations highlight the need to adjust for age in order to assess general relations between SD BOLD and behavioral measures in our sample.

**Fig. 1.**
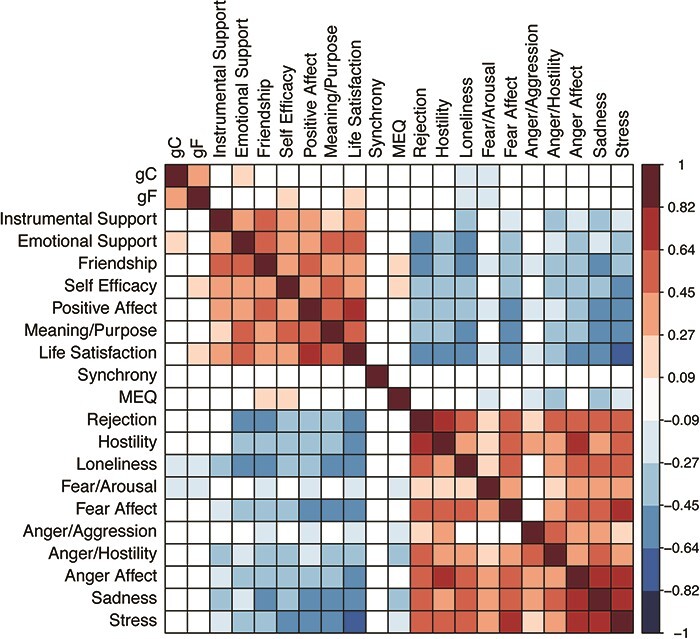
The correlations among all the behavioral variables are shown: positive correlations are indicated by red and negative correlations by blue. All correlations shown are significant at *P* < 0.05. Correlation magnitude is indicated by the color shade (lighter to darker) as indicated by the scale on the right side of the figure.

Correlations among the age-adjusted behavioral variables are shown in Figure 1. The two cognitive composites were significantly correlated with one another. Although these two composites were not strongly correlated with the socioemotional measures, they tended to be positively correlated with the positive emotion measures and negatively correlated with the negative emotion measures. Most of the correlations within the group of positive emotional measures and within the group of negative emotional measures were significantly positive, whereas the majority of the correlations between the positive and negative emotional measures were significantly negative. Synchrony was not correlated with any of the other scores; in general, MEQ scores were correlated positively with the positive socioemotional measures and negatively with the negative socioemotional measures.

### PLS analysis

**Fig. 2.**
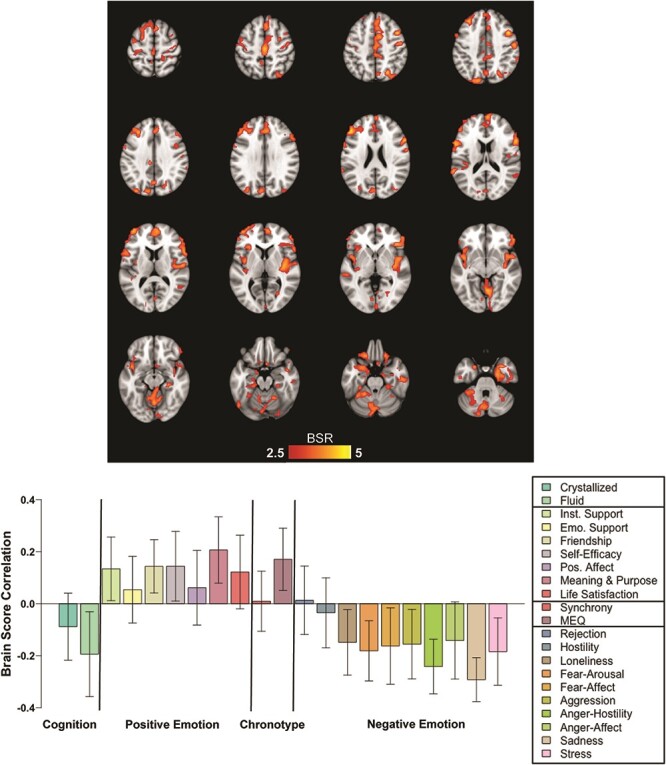
The voxels with above-threshold contributions (as indicated by the BSR) to the pattern of brain–behavior correlations identified by PLS are shown in the spatial map at the top. The correlations between BOLD-SD and the behavioral scores are shown in the graph, along with the CIs for each correlation. Positive correlations indicate that increasing BOLD-SD is associated with higher scores for that measure. Negative correlations indicate that increasing BOLD-SD is associated with lower scores for that measure.

**Fig. 3.**
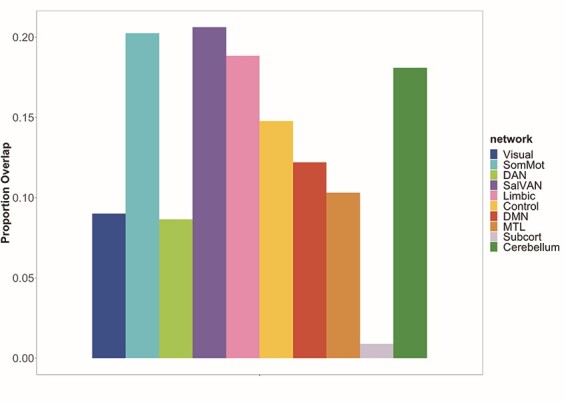
This figure shows how each network/region contributes to the pattern of correlation between BOLD-SD and the behavioral measures. The graph shows, for each network or region, the proportion of voxels (relative to the total number of voxels in each network or region) that showed a significant relation between BOLD-SD and the behavioral measures (i.e. had above-threshold BSRs). The seven networks were defined from functional connectivity analyses, whereas the MTL, subcortical and cerebellar regions were defined anatomically. Abbreviations: Visual = visual network; SomMot = somatomotor network; DAN = dorsal attention network; SalVAN = salience/ventral attention network; Limbic = limbic network; DMN = default mode network; MTL = anatomically defined medial temporal regions; Subcort = anatomically defined subcortical regions; Cerebellum = anatomically defined cerebellum region

The analysis of BOLD-SD and behavioral scores identified a single LV (*P* = 0.001, 58% covariance accounted for; Figure 2). Regions with above-threshold contributions to the pattern of correlations between BOLD-SD and performance on the cognitive measures included precentral, postcentral, inferior frontal, insula and cerebellum bilaterally (Table 2). Medial temporal regions also contributed to this spatial pattern, involving the bilateral parahippocampal gyrus and left amygdala. In all above-threshold voxels, there was a negative correlation between BOLD-SD and gF, as well as negative correlations between BOLD-SD and most of the negative socioemotional variables (Figure 2). That is, greater BOLD-SD in these regions was associated with lower gF and lower negative emotion scores. In contrast, scores on four of the positive socioemotional measures (instrumental support, friendship, self-efficacy and meaning & purpose) and MEQ scores were associated with greater BOLD-SD. Synchrony and gC did not contribute to this pattern of effects. In terms of brain networks, the strongest contributions to the spatial pattern were from Som-Mot, SAL/VAN, LIM and CON networks, and the Cerebellum (Figure 3). The DMN and MTL showed somewhat smaller contributions.

**Table 2.** Cluster maxima for regions showing correlations between behavioral measures and BOLD-SD

Region/gyrus	Hem	BA	*X* (mm)	*Y* (mm)	*Z* (mm)	BSR
Orbitofrontal	L	11	−20	28	−24	5.03
Orbitofrontal	R	11	24	28	−24	3.81
Middle frontal	L	9	−48	36	24	4.70
Middle frontal	L	6	−24	16	64	3.97
Middle frontal	R	6	44	12	44	4.57
Inferior frontal	L	44	−56	8	16	4.16
Inferior frontal	R	45	60	16	20	4.51
Medial frontal	R	9	8	56	8	3.93
Precentral	L	6	−32	−4	64	3.31
Paracentral	M	6	0	−20	52	4.32
Postcentral	R	3	56	−12	44	3.73
Subcallosal	R	25	4	12	−20	4.03
Posterior cingulate	L	31	−8	−28	36	3.86
Superior temporal	L	22	−64	−44	20	4.04
Superior temporal	R	38	32	8	−32	4.41
Middle temporal	L	21	−56	−28	−4	3.14
Inferior temporal	L	20	−48	−12	−32	3.50
Parahippocampus	L	36	−20	−28	−20	3.27
Parahippocampus	R	35	28	−28	−12	3.82
Amygdala	L		−12	−4	−24	3.52
Superior occipital	L	19	−36	−84	36	4.02
Superior occipital	R	19	36	−84	32	3.23
Inferior occipital	R	18	24	−88	−20	3.08
Cuneus	M	7	0	−68	36	3.47
Fusiform	L	19	−52	−68	−16	3.37
Lingual	R	19	28	−68	4	3.13
Precuneus	R	7	24	−68	48	4.45
Superior parietal	R	5	28	−40	68	3.70
Supramarginal	R	40	64	−40	40	3.47
Insula	L	13	−44	8	−12	3.71
Insula	R	13	40	−12	3	3.96
Cerebellum	L		−12	−52	−56	3.34
Cerebellum	R		28	−40	−48	4.39
Cerebellum	M		0	−60	−8	4.82

Abbreviations: Hem = hemisphere; BA = Brodmann’s area; *X* = R/L coordinate; *Y* = anterior/posterior coordinate; *Z* = superior/inferior coordinate; R = right; L = left; M = midline.

**Fig. 4.**
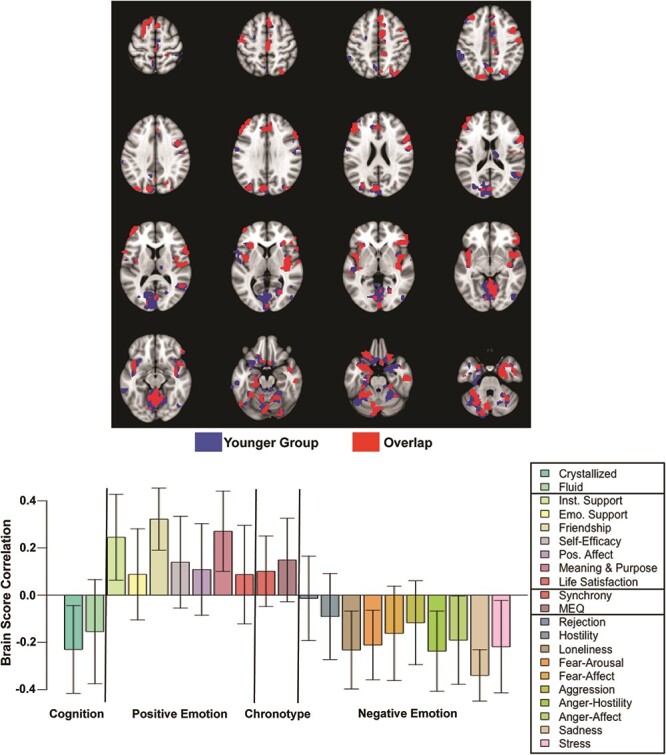
The voxels with above-threshold contributions to the pattern of brain–behavior correlations identified by PLS in the younger group (below the median age) are shown in the spatial map at the top. The voxels that were above threshold only in the younger group are shown in blue, and voxels from this analysis that overlap with those from the analysis of the full sample (seen in Figure 2) are shown in red. The correlations between BOLD-SD and the behavioral scores are shown in the graph, along with the CIs for each correlation. Positive correlations indicate that increasing BOLD-SD is associated with higher scores for that measure. Negative correlations indicate that increasing BOLD-SD is associated with lower scores for that measure.

Contrary to our predictions, there were no above-threshold voxels with positive correlations between BOLD-SD and performance on the two cognitive composites. To determine if this result was due to the inclusion of older adults, and the need for adjusting for age in both behavioral and brain datasets, we carried out the same PLS analysis in a subsample of our participants below the median age, i.e. all those below the age of 49 years (*N* = 78). In this analysis, we used the BOLD-SD data that had not been adjusted for age (only for the mean FD) and the scores for the behavioral data that had not been adjusted for age. This analysis resulted in a single LV (*P* = 0.009, accounting for 58.8% of covariance, Figure 4), with a pattern of effects across the behavioral variables that closely resembled that of the pattern seen in the full sample. Although these effects were weaker than those seen with the full sample, there were positive correlations between BOLD-SD and the positive socioemotional variables and MEQ scores, as well as negative correlations between BOLD-SD and the cognitive composites and negative emotional scores. Finally, the spatial pattern of correlations seen in the younger group (blue voxels in Figure 4) was highly overlapping with the regions identified in the full sample (red voxels in Figure 4).

## Discussion

In this study, we examined the relations between resting brain signal variability and cognitive and socioemotional characteristics, including chronotype. Consistent with our hypotheses, we found that better socioemotional function and morning chronotype were associated with greater resting BOLD-SD in a number of brain networks, notably those involved in cognitive control, attention, motor function and emotional regulation. This result is consistent with prior work showing that greater task-related BOLD-SD is associated with better and more consistent cognitive performance. Surprisingly, we found no regions where greater BOLD-SD during rest was accompanied by better scores on cognitive measures but instead found that reduced BOLD-SD was related to better cognitive performance on the fluid composite. This result contrasts with task-related findings in general but is consistent with our prior work showing a similar relation between reduced sensorimotor BOLD-SD during task and better working memory performance in this sample (Rieck *et al.*, 2022).

Although much research has shown a positive correlation between brain signal variability and cognitive performance when both are measured during a scanned task (Garrett *et al.*, 2011; Waschke *et al.*, 2021), the relation between resting variability and performance on standard cognitive measures such as gF and gC has not been as well studied. Our results showed a negative relation between resting BOLD variability and fluid cognition, and no relation between BOLD-SD and crystallized cognition, once age has been taken into account. Interestingly, we found a similar pattern just in our younger adults (below age 49 years), indicating that this effect was not an artifact of regressing age out of our data. Therefore, our results suggest that if resting BOLD-SD reflects the dynamic range of spontaneous brain function, then individual differences in flexible cognitive performance may be facilitated by greater stability at rest in a number of networks. Using cognitive constructs similar to those examined here, an earlier study (Garrett *et al.*, 2021) found that increased resting BOLD-SD was associated with higher scores on gF, the opposite of what we found, but was not significantly related to gC, similar to our result. However, age was also included as a variable in the model and the strongest correlation was a negative one between BOLD-SD and age. Since gF is typically negatively correlated with age, this suggests that the pattern of BOLD-SD and behavior correlation seen in this earlier study may have been influenced by the relation between BOLD-SD and age. Taken together with our finding that greater BOLD-SD is associated with lower gF when the effect of age is removed from both the brain images and the behavioral data, these results suggest that (I) the relation between BOLD-SD and age is strong in general and may influence other effects if not accounted for and (II) the relation between BOLD-SD and cognition is complex and may reflect both positive and negative effects depending on the particular experimental condition (e.g. rest *vs* task).

We found an association between greater BOLD-SD and better socioemotional function, such as greater life satisfaction and self-efficacy. This is consistent with our hypothesis that increased brain signal variability reflects more flexibility and greater dynamic range of brain activity and thus facilitates one’s ability to adapt to the environment, including the socioemotional milieu. Similarly, we found a relation between increased BOLD-SD and morningness chronotype, which is associated with better emotional regulation and positive affect (Ottoni *et al.*, 2012; Watts and Norbury, 2017; Lunsford-Avery *et al.*, 2019; Tan *et al.*, 2020). This effect appears to be related to chronotype *per se* and not to any discrepancy between chronotype and time of scanning because the synchrony measure, which would account for any such discrepancy, was not robustly related to BOLD-SD. The common correlational pattern of increased variability in the amygdala and regions supporting cognitive control suggests that flexible resting BOLD-SD in these areas may be one mechanism underlying better socioemotional adaptation in those with a morning chronotype. In particular, the recruitment of the amygdala and other regions involved in emotion processing and regulation, such as the insula and ventral frontal regions, provides further evidence of the importance of these brain areas for successful emotional function. Specifically, the area of maximum effect in the amygdala was in the medial part of this region (Table 2) although the lateral aspect of the amygdala was also above threshold (Figure 2). Recent work has found that the medial amygdala is strongly connected to the ventromedial PFC and participates in a network associated with social affiliation, whereas the lateral amygdala is more strongly connected to the lateral PFC and supports the perception of social signals (Bickart *et al.*, 2012; de Gelder *et al.*, 2014; Jiang *et al.*, 2019). Here, we find that both segments of the amygdala, as well as medial and lateral portions of the ventral PFC, contribute to the positive associations among BOLD-SD, positive socioemotional scores and morning chronotype. This result suggests that BOLD variability in the medial amygdala and ventromedial PFC may facilitate social affiliations with other people, whereas BOLD variability in the lateral amygdala and lateral PFC may support adaptive perception of social stimuli. This interpretation would clearly need to be supported with additional data, but our results indicate that linking BOLD variability in specific amygdala networks to different aspects of socioemotional function could be a fruitful avenue for future research.

In conclusion, we have shown that resting BOLD-SD is related to both cognition and measures of socioemotional function. Taken together with our earlier finding of correlations between task-related BOLD-SD and working memory performance in these participants (Rieck *et al.*, 2022), this work indicates that the relation between brain signal variability and behavioral performance has both short-term and longer-term characteristics. That is, spontaneous BOLD-SD during rest can be thought of as relatively stable, reflecting variability that may be somewhat independent of specific cognitive demands, but related to cognitive and socioemotional characteristics of individuals that are also relatively stable over time. In addition, BOLD-SD during externally driven experimental tasks likely reflects variability that can be modified as a function of the particular cognitive demands required by the specific task at hand and thus is related to cognitive performance during that given task (for a similar idea regarding BOLD functional connectivity, see Geerligs *et al.*, 2015). Note that this does not exclude the possibility that BOLD variability obtained during a task could be related to behavior measured outside of the scanner on a different task and/or day (Millar *et al.*, 2020; Rieck *et al.*, 2022). Future work should determine whether correlations between task-related BOLD-SD and cognitive performance outside the scanner are due to links between task-specific variability and more general cognitive characteristics or between the latter and underlying intrinsic activity present in the background of task-related fMRI measures. Although the finding that increased resting BOLD-SD is associated with higher positive emotional scores and lower negative emotional scores fits with our hypothesis that more brain variability should facilitate optimal function, it is unclear why increased resting BOLD-SD should be associated with lower fluid intelligence. We would have expected a positive association between gF and BOLD-SD. Nonetheless, the negative association that we observed between gF and BOLD-SD may be partially explained by a previous study that used structural equation modeling to examine gF and emotional function in a large sample of 1400 undergraduates (Simonet *et al.*, 2021). This study showed a relation between higher gF and more negative feelings, as well as an association between higher gF and lower positive feelings. Although gF was not in general positively related to negative emotion scores in our sample, there may nevertheless be a common sensitivity of both gF and negative emotions to respond similarly to greater stability of BOLD-SD. Further research is needed to test this idea directly. Finally, we focused on brain–behavior relations independent of age, but it is possible that age would influence the correlations reported here. In particular, age influences might be seen in how BOLD variability in the DMN is related to cognitive or socioemotional factors, given the prominent age differences noted in functional connectivity of this network (Andrews-Hanna *et al.*, 2007; Damoiseaux *et al.*, 2008; Chan *et al.*, 2014; Grady *et al.*, 2016), including in the context of socioemotional cognition (Grady and Garrett, 2018; Fareri *et al.*, 2022).

## Data Availability

The data statement is in the section titled Brain variability measures.
